# Correction to: Case series of sebelipase alfa hypersensitivity reactions and successful sebelipase alfa rapid desensitization.

**DOI:** 10.1002/jmd2.12120

**Published:** 2020-05-07

In this paper,^1^ the footnote in Table 2 incorrectly states the concentration of drug in the 2 solutions used for the desensitization.

The corrected footnote for Table 2 is as follows:

Solution 1:1000 mL volume; 20 mg/1000 mL concentration.

Solution 2:100 mL volume; 20 mg/100 mL concentration.
